# Aspiration versus retention ultrasound-guided ethanol sclerotherapy for treating endometrioma: A retrospective cross-sectional study

**DOI:** 10.18502/ijrm.v13i11.7960

**Published:** 2020-11-22

**Authors:** Abbas Aflatoonian, Nasim Tabibnejad

**Affiliations:** Research and Clinical Center for Infertility, Yazd Reproductive Sciences Institute, Shahid Sadoughi University of Medical Sciences, Yazd, Iran.

**Keywords:** Endometrioma, Ethanol, Sclerotherapy, In vitro fertilization, Pregnancy rate.

## Abstract

**Background:**

Endometrioma is a common high-recurrence gynecological disease that affects infertility. Surgical resection using laparotomy or laparoscopy is applied as a standard treatment. Moreover, sclerotherapy is reported to be effective as a non-invasive method for treating endometrioma.

**Objective:**

To evaluate whether the ethanol retention or aspiration after sclerotherapy improve pregnancy outcome in infertile women with endometrioma.

**Materials and Methods:**

In a retrospective study, hospital records of 43 women with recurrent or bilateral endometrioma who had been undergone transvaginal ultrasound sclerotherapy were reviewed. They were selected to receive either ethanol for 10 min, ethanol injection, irrigation, and then aspiration or total retention without aspiration based on the surgeon's decision. The participants were followed-up for 3, 6 and 12 months for natural or artificial conception as well as for cyst recurrence.

**Results:**

Chemical pregnancy was positive in 52% of the women in the aspiration group and 53.8% in the retention group. Ongoing pregnancy (44% vs 46.2%, p = 0.584) and live birth (40% vs 46.2%, p = 0.490) were reported marginally higher in the retention group compared with the aspiration group, and the differences were not statistically significant. Moreover, the recurrence rate were found to be 48.1% and 37.5% in the aspiration and retention groups, respectively (p = 0.542). The cysts size in the retention group was significantly correlated to the recurrence rate.

**Conclusion:**

Both the aspiration and left in situ of ethanol 95% sclerotherapy have the similar impact on the treatment of ovarian endometrioma regarding pregnancy and recurrence rate. However, larger randomized studies with strict inclusion criteria are needed.

## 1. Introduction

Endometriosis is defined as the presence of endometrial glands and stroma outside the uterine cavity, frequently found in the ovary in 17-44% of women with endometriosis (1). Ovarian endometrioma is an ovarian cyst which is bound by endometrial tissue and holds menstrual debris. Endometrioma is displayed by dysmenorrhea (2), pelvic pain, and subfertility (3). The conventional management of these ovarian cysts is surgery and the most common surgical process is laparoscopic cystectomy. Several reports have indicated that surgical treatment have a detrimental effect on ovarian reserve (4-6) due to the removal of healthy tissue next to the cyst wall (7, 8), besides excessive ovarian coagulation (5). Therefore, there are controversies regarding the surgical management (9-13) along with changes of attitudes toward a less invasive approach especially in the cases of infertility. On the other hand, medical therapy has been widely investigated in the treatment of ovarian endometrioma. Oral contraceptive pills, progestins, gonadotropin-releasing hormone agonists (14) as well as aromatase inhibitors (15) are effective to recover symptoms, reduce cyst size, and decrease the risk of post-surgery occurrence (16). However, the majority of cases show symptom reappearance by withdrawing medical treatment (17). Alternatively, some studies have reported the effectiveness of sclerotherapy on the reduction of endometrioma recurrence rate (18-20). Ethanol sclerotherapy in combination with transvaginal ultrasound aspiration significantly reduce one-year recurrence rate among women with recurrence cyst (18) and could be considered as an effective alternative to surgery in the management of endometrioma (21). Discrepancy in the results of the studies investigating the effect of ethanol sclerotherapy on the recurrence endometrioma may be due to the difference in ethanol concentration and volume in addition to the number of the cysts and cyst size (22). Furthermore, it is assumed that varied treatment time may also affect the total recovery rate of endometrioma (21, 23).

Therefore, in this study, we aimed to extent our previous practice (24) to compare the different time of ethanol exposure with regard to the risk of recurrence and reproductive outcome in ovarian endometrioma.

## 2. Materials and Methods

The current study was a retrospective review of hospital data records of 43 women referred to the Yazd Research and Clinical Center for Infertility between March 2011 and April 2017.

### Subjects

Women with Follicle stimulating hormone (FSH) < 10, aged > 20 but < 39 yr, postsurgical recurrent endometrioma or bilateral endometrioma without surgical indication were included in this study. They had been offered transvaginal ultrasound sclerotherapy with 95% ethanol. The diagnosis of endometrioma was confirmed through sonography and the result of previous laparotomy/laparoscopy. Additionally, cysts with features indicative of dermoid cysts, women with ascites, an abnormal coagulation test, history of gynecologic cancer, refusal to participation in the study, and those who were lost to follow-up had been excluded.

### Procedure

In this retrospective study, women were experimentally selected to receive either ethanol for 10 min, ethanol injection, irrigation, and then aspiration (aspiration group) or total retention without aspiration (retention group) based on the surgeon decision. In the operating room, pentazocine (Ampulla, 30 mg/ml, Tolidaru, Iran) and pethedine (Ampulla, 50 mg/ml, Kaspian, Iran) had been administered as analgesic agents to them. The site had been prepped and draped in a sterile method. Under vaginal ultrasonographic guidance (Honda 4000, Japan), a needle (three-way needle, Wallace, UK) was inserted into the endometrioma and the contents were aspirated. The cyst cavity was irrigated with a physiological saline till the fluid was clear in color. Next, the cyst cavity was infused with 98% ethanol for 10 min and then removed. In women prescribed with ethanol retention, the ethanol was left in the cyst without aspiration. The volume of ethanol instilled was equal to two-third of the volume aspirated from the endometrioma and was calculated by 80%. The drained fluid was then sent for a cytological examination to identify any atypical cells. Cefixim 400 mg/day was started as a prophylactic antibiotic before and after sclerotherapy for 1 wk. The women were followed up for 3, 6, and 12 months for natural or artificial conception, as well as cyst recurrence. Participants' age, duration of infertility, cyst size and recurrence rate, and the ART outcome were compared between the groups. Recurrence was defined as a cyst diameter of > 3 cm. While chemical pregnancy was defined as positive βhCG 14 days after the embryo transfer, ongoing pregnancy was defined as existence of fetal heart activity on ultrasound after 12 wk.

### Ethical consideration

The study was approved by the Ethics Committee of Yazd Reproductive Sciences Institute, Shahid Sadoughi University of Medical Sciences; IR.SSU.RSI.REC.1398.009.

### Statistical analysis

Data were analyzed using the Statistical Package for the Social Science version 20 for Windows (SPSS Inc, Chicago. IL, USA). The differences between continuous variables with normal distribution were calculated using the Student *t* test. The Chi-square test was used to compare categorical variables, and p < 0.05 was set as the level of significance for this study.

## 3. Results

**Figure 1:**
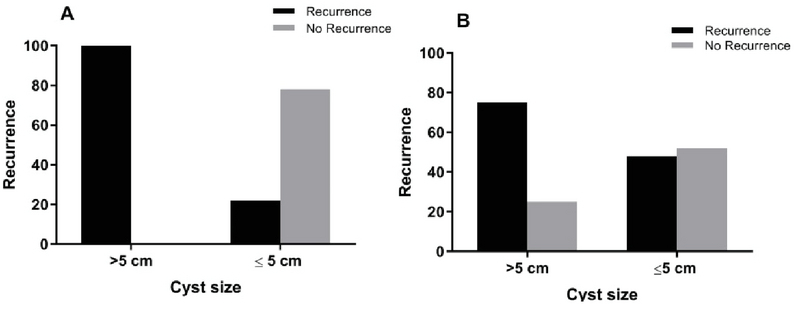
Endometrioma recurrence rate with regard to cyst size: (A) Retention group; significant differences in recurrence rate between the cyst size of > 5 cm and the cyst size of ≤ 5, p = 0.021, Chi-square test. (B) Aspiration group.

The mean age of the patients was 31.47 ± 4.93 yr, the mean infertility duration was 7.95 ± 4.08 yr, and the mean cyst size was 4.52 ± 1.27 cm. No surgical complication occurred for any patient. No atypical cell was observed in the cytology reports of aspirated cysts fluid. The characteristics of both aspiration and retention group are presented in table I. Moreover, two groups were similar in terms of age, duration of infertility, and cyst size. Two women (7.4%) in the aspiration group and three (18.8%) in the retention group were lost to follow-up. Therefore, 25 women in the aspiration and 13 in the retention groups were analyzed for the pregnancy outcome and recurrence rate. With regard to pregnancy outcome, 52% of the women in the aspiration and 53.8% in the retention group were detected with positive chemical pregnancy. Ongoing pregnancy (44% vs 46.2%) and live birth (40% vs 46.2%) were reported marginally higher in the retention group compared with the aspiration group, and the differences were not statistically significant (Table II). Among the 13 pregnant women in the aspiration group, 6 women (46.2%) had conceived through in vitro fertilization (IVF) and 7 women (53.8%) became pregnant spontaneously. However, in the retention group, 57.1% of the women achieved pregnancy through assisted reproduction technique and 42.9% reached spontaneous conception. The recurrence rates were found to be 52% and 46.2% in the aspiration and retention groups, respectively (p = 0.500) (Table II). The cysts size in the retention group significantly correlated to the recurrence rate. All cysts > 5 cm showed a recurrent form and 77.8% of cysts with a diameter of ≤ 5 cm did not recur (p = 0.021) (Figure 1).

**Table 1:** Characteristics of aspiration versus retention groups


**Variables**	**Aspiration (n = 27)**	**Retention (n = 16)**	P-value*
**Age (yr) **	31.70 ± 4.79	31.06 ± 5.31	0.902
**Duration of infertility (yr) **	8.33 ± 4.49	7.31 ± 3.30	0.371
**Cyst size (cm) **	4.38 ± 1.20	4.76 ± 1.38	0.870
Data are presented as Mean ± SD; *student *t* test			

**Table 2:** Pregnancy outcome and recurrence rate in aspiration versus retention groups


**Variables**	**Aspiration (n = 25)**	**Retention (n = 13)**	P-value*
**Chemical pregnancy **	13 (52)	7 (53.8)	0.593
**Ongoing pregnancy**	11 (44)	6 (46.2)	0.584
**Live birth **	10 (40)	6 (46.2)	0.490
**Recurrence rate **	13 (52)	6 (46.2)	0.500
Data are presented as number (%); *Chi-squared test			

## 4. Discussion

In this study, we found that both aspiration and left in situ of ethanol 95% have the same effect on the treatment of ovarian endometrioma in terms of pregnancy rate as well as the recurrence rate. The preliminary treatment approach was cyst aspiration, followed by ethanol injection either removed after 10 min or retention.

Ethanol sclerotherapy is a good alternative policy to avoid detrimental effects of laparoscopic cystectomy on ovarian function (18, 25). Andre and colleagues *et al* reported a 20% pregnancy rate after ethanol sclerotherapy of ovarian endometrioma subsequent to ovarian stimulation with GnRh agonist protocol (26). In our previous clinical trial, we enrolled 40 women with recurrent endometrioma for an IVF cycle; 20 patients randomly underwent ethanol sclerotherapy. The result showed no significant differences in the implantation and fertilization rates as well as the clinical pregnancy rate in both groups (24). Likewise, Lee and colleagues found similar results in terms of implantation and clinical pregnancy rates between three groups of patients with endometrioma (surgical resection, aspiration with ethanol sclerotherapy, and the group with no intervention (27).

With regard to the effect of treatment time on the pregnancy, in contrast to our findings, Chang and colleagues found that the pregnancy rate was significantly higher in patients who received longer treatment (more than 7 min and left ethanol in situ) than in the patients with shorter treatment time (0-6 min) (23). However, the total pregnancy rate after ethanol sclerotherapy in patients undergoing IVF ranged from 20-57%, but the result of a systematic review and meta-analysis revealed that the pregnancy rate was similar after endometrioma sclerotherapy compared with patients who underwent laparoscopic cystectomy, and also those with no intervention (22). Another study that evaluated the effect of ethanol sclerotherapy duration on the treatment of ovarian endometrioma, compared the time of sclerotherapy up to 10 min and ethanol retention. The authors did not report pregnancy rate but indicated that one-year recurrence rate was significantly lower (13%) in patients treated with in situ ethanol retention (18). Otherwise a high cyst recurrence rate was found in both groups with no significant difference between ethanol washing and ethanol retention group. Also, Noma and Yoshida reported a 9.1% recurrence rate using the treatment with ethanol for ≥ 10 min vs. 62.5% recurrence rate with instillation for < 10 min (28). Likewise, in the endometrioma treatment using 95% ethanol, the recurrence rate was found to be 13.3% in the retention group versus 32.1% in the irrigation group (29).

In our previous study, endometrioma was relapsed in 20% of women after 10 min of sclerotherapy (24). Similarly, two other studies reported a cyst recurrence rate of 8% and 12% in patients with 10 and 15 min of sclerotherapy with ethanol, respectively (21, 30). Moreover, Castellarnau *and colleagues* described the recurrence rate of 22.7% in patients who underwent ethanol sclerotherapy compared with those who underwent simple aspiration (72.7%) (31). It is documented that simple ultrasonography aspiration of endometrioma lead to a high recurrence rate by the range of 53-97.6% (31, 32). Nevertheless, when ethanol sclerotherapy was added to the procedure, both the recurrence rate and following infertility were decreased (20, 21, 28, 33, 34). The results of a meta-analysis revealed that the rates of endometrioma recurrence were 0-13.3% when ethanol was left in situ and 0-62.5% while it was used for washing up to 15 min. Therefore, the risk of cyst recurrence was significantly lower in women who were treated with the use of prolonged ethanol washing than those treated with the use of short ethanol washing (22). In contrast, another meta-analysis stated that endometrioma aspiration followed by sclerotherapy does not seem to significantly reduce the likelihood of recurrence. However, it should be noted that this review analyzed that studies used different sclerosing agents together with ethanol (35).

We also found that the cyst size in the ethanol retention group has a positive correlation with the recurrence rate. It has been shown previously that a larger cyst diameter is significantly associated with recurrence (31) and that women with cyst size ≤ 5 cm experience a higher recovery rate (23). Additionally, Andre *and colleagues* stated that ovarian cysts with a mean diameter of 4.7 cm did not recur after aspiration (26). It is confirmed that larger cysts have thick multiple septa and several cavities and hold more pathologic fillings; therefore, a larger area of endothelial lining is involved during the manipulation of these cysts. Mentioned features of large cysts decrease the ethanol efficacy and lead to incomplete procedures and higher failure rate (23).

## 5. Conclusion

In conclusion, our result showed that sclerotherapy using both aspiration and left in situ of ethanol 95% have a similar impact on the treatment of ovarian endometrioma regarding the pregnancy and recurrence rates. However, larger randomized studies with strict inclusion criteria are necessary to evaluate the effect of endometrioma sclerotherapy on bilateral cyst and improvement of ART outcome.

##  Conflict of Interest 

The authors declare that there is no conflict of interest.
